# Modeling the Responses to Resistance Training in an Animal Experiment Study

**DOI:** 10.1155/2015/914860

**Published:** 2015-01-28

**Authors:** Antony G. Philippe, Guillaume Py, François B. Favier, Anthony M. J. Sanchez, Anne Bonnieu, Thierry Busso, Robin Candau

**Affiliations:** ^1^INRA, UMR866 Dynamique Musculaire et Métabolisme, Université de Montpellier, 34000 Montpellier, France; ^2^Département STAPS, Laboratoire Européen Performance Santé Altitude, Université de Perpignan, EA 4604, Via Domitia, 66120 Font-Romeu, France; ^3^Laboratoire de Physiologie de l'Exercice, Université de Lyon, 42000 Saint-Etienne, France

## Abstract

The aim of the present study was to test whether systems models of training effects on performance in athletes can be used to explore the responses to resistance training in rats. 11 Wistar Han rats (277 ± 15 g) underwent 4 weeks of resistance training consisting in climbing a ladder with progressive loads. Training amount and performance were computed from total work and mean power during each training session. Three systems models relating performance to cumulated training bouts have been tested: (i) with a single component for adaptation to training, (ii) with two components to distinguish the adaptation and fatigue produced by exercise bouts, and (iii) with an additional component to account for training-related changes in exercise-induced fatigue. Model parameters were fitted using a mixed-effects modeling approach. The model with two components was found to be the most suitable to analyze the training responses (*R* ^2^ = 0.53; *P* < 0.001). In conclusion, the accuracy in quantifying training loads and performance in a rodent experiment makes it possible to model the responses to resistance training. This modeling in rodents could be used in future studies in combination with biological tools for enhancing our understanding of the adaptive processes that occur during physical training.

## 1. Introduction

Adaptations to training are related to the amount of work performed during the exercise sessions. The sum of these inputs yields increases and decreases in performance capacity because both adaptation and fatigue are produced by exercise bouts. Systems models have been developed to quantify these antagonistic effects of physical exercise on human performance. In 1975, Banister et al. [1] proposed the first and most frequently used model, which includes two components in order to distinguish the adaptations and fatigue that occur with training. A simpler model with only one component was also proposed to analyze the biological responses induced by endurance training [2]. The most complex model is an extension of that proposed by Banister et al., in which the response to a single exercise depends on past training [3]. Using such models with data from animal experiments would offer the opportunity to go beyond the simple quantification of the relationship between the amount of exercise training and performance and would thereby improve our knowledge about the nature of the adaptive processes that take place during training.

Modeling training effects in rodents presents several advantages over models in athletes. Animal models allow the measurement of training effects for a broad range of training situations, loads, and intensities, which would be unethical in athletes. Moreover, the training load and performance can be controlled with high precision, especially in the context of resistance exercise (RE) on a climbing ladder. This precision enables researchers to capture small details of the training process and, ultimately, to optimize the structure of the model itself. Rodent models authorize greater invasiveness, yield more biological information, and therefore provide greater insight into the adaptive processes that occur during training, particularly regarding the link between the adaptive cell mechanisms and training effects. In addition, the animal model could reduce the sources of variability in response to training compared with a human model. The interindividual variability is naturally decreased in animals with the same genetic background. Obviously, parameters external to training (nutrition, sleep quality, fatigue related to activities other than training, etc.) are controlled in animals as opposed to humans. This homogeneity in the responses to physical exercise in animals allows us to take advantage of mixed-effects modeling to analyze the responses of a group of animals, taking interindividual variability into consideration. When repeated measurements are made on several related statistical units, mixed-effects modeling allows a more robust estimation of model parameters than using only available individual data [4–6]. The single-individual model has generally been used in human studies, with the exception of one work in which the mixed-effects model was applied to a group of elite swimmers [7].

Among the training programs, RE is particularly suitable for animal studies because RE is associated with high gains in performance, muscle strength, and muscle fiber cross-sectional area. RE is characterized by exercise performed between 60% and 80% of the maximum load, and several experimental models have been developed to evaluate muscle and physical performance in response to RE. In rats, voluntary exercise based on ladder climbing activity has been shown to induce muscle hypertrophy, changes in muscle typology, and increased force and power output [8]. One of the first studies using ladder climbing as a model of resistance training [9] showed that, after 26 weeks of resistance training, the trained rats were able to climb 40 cm while carrying up to 140% of their body mass, without changes in the ratio between body and muscle (EDL and soleus) mass, in comparison with controls. More recently we found that rats could climb 1 meter while carrying 150% of their body mass after 4 weeks of resistance training, in association with hypertrophy of 48% of fiber IIx in FDP muscle [10]. After 8 weeks, the rats could lift up to 210% of their body mass. Another study [11] demonstrated a 287% increase in the maximal amount of body weight that the animals could carry after 8 weeks of training (3 sessions a week).

RE model offers the opportunity to quantify both training work and performance in animal with a great accuracy. Thus, the twofold aim of the present study was to (i) test whether the systems models used to describe the training response in athletes could be applied in rats and (ii) verify the applicability of the mixed-effects model in animals with the same genetic background in order to improve the statistical strength of the training response model.

## 2. Methods

### 2.1. Animals and Experimental Design

#### 2.1.1. Ethics Statement

This study was approved by the Committee on the Ethics of Animals Experiment of Languedoc Roussillon in accordance with the guidelines from the French National Research Council for the Care and Use of Laboratory Animals (permit number: CEEA-LR-1069).

#### 2.1.2. Animal Model

Eight-week-old Wistar Han rats (277 ± 15 g; *n* = 11) obtained from Charles River Laboratories (L'Arbresle, Rhône, France) were housed at a constant room temperature and humidity and maintained in a 12 : 12 h light-dark cycle. They had access to standard rat chow (A04, Scientific Animal Food & Engineering, Augy, France) and water* ad libitum*.

#### 2.1.3. Resistance Training Protocol

The rats underwent 4 weeks of progressive resistance training. The exercise consisted of climbing a 1-meter-high homemade ladder inclined at 85° ten times. The ladder was adapted from the apparatus of Lee et al. [12]. Training sessions were held in the afternoon, five times a week. A cloth bag containing weights was attached to the base of the tail with tape. Three days before training, the rats were familiarized with the apparatus by climbing it twice with 50% of body weight. In accordance with the protocol proposed by Begue et al. [10], the initial weight attached to the tail was 50% of the rat body weight and was increased progressively until 150% after 4 weeks (Table 1). Each training session consisted in one set of 10 repetitions with 2 min rest between trials. All rats were able to perform ten climbs per training session. Rats from the same cage were trained together. Precisely, rats were placed on a platform on the top of the ladder and one of them was put on the floor at the base of the ladder. The working rat quickly joined its congeners spontaneously.

### 2.2. Training and Performance Quantification

Training work (TW in J) was calculated as the potential work developed during the training sessions:

(1) $$\begin{array}{c} \text{TW}=\left(m_{\text{load}}+m_{\text{rat}}\right)\cdot g\cdot \Delta h\cdot N, \end{array}$$

where mass (*m*) is expressed in kg, *g* is the constant of the gravity on earth expressed in m·s^−2^, *h* is the distance climbed in m, and *N* is the number of repetitions.

Performance was the power output developed during the full climbing session, computed as the work done against gravity (TW) divided by total climbing time (s) and expressed in W:

(2) $$\begin{array}{c} \text{Performance}=\frac{\text{TW}}{\text{time}}. \end{array}$$

Each climb generally lasted between 3 and 25 s depending on the load carried by the rats.

### 2.3. Modeling of the Training Effects

#### 2.3.1. Basic Frameworks

Since the original work of Banister and coworkers [1], systems modeling has been used to analyze the adaptations to physical training in subjects enrolled in controlled experiments or in athletes in real-life situations [13, 14]. This approach considers the body as a system whose output is the performance varying with the amounts of training ascribed to input. Systems theory allows the analysis of a dynamical process using abstraction from mathematical models. A system is characterized by at least one input and one output, and the system behavior is characterized by a transfer function *H*(*t*, *θ*) relating output at a given time to previous inputs. Assuming the formulation of the transfer function, the set of parameters characterizing a subject's behavior (noted *θ*) is estimated by fitting the model output to the actual data. The number of parameters which can be introduced in the model is limited by the precision of the data that can be collected to quantify training input and performance output. An analysis of the goodness-of-fit is needed to test the statistical significance of the model, especially to compare models differing in complexity, that is, the number of equations and related parameters giving the degrees of freedom of the competing models (df).

The transfer function *H*(*t*, *θ*) gives the model performance at time *t* by using the product of convolution as follows:

(3) $$\begin{array}{c} p\left(t\right)=p\left(0\right)+w\left(t\right)*H\left(t,\theta \right), \end{array}$$

where *p*(0) is the initial performance and the product of convolution is defined by

(4) $$\begin{array}{c} w\left(t\right)*H\left(t,\theta \right)=\overset{t}{\underset{0}{\int }}w\left(s\right)\cdot H\left(t-s,\theta \right)ds. \end{array}$$

The discretization of (2) gives

(5) $$\begin{array}{c} p\left(n\Delta t\right)=p\left(0\right)+\overset{n-1}{\underset{i=1}{\sum }}w(i\Delta t)\cdot H\left(\left(n-i\right)\Delta t,\theta \right), \end{array}$$

where *t* = *n*Δ*t* and *w*(0) is assumed to be equal to 0. Fixing the value of Δ*t* to 1 day led us to consider *w*(*t*) as a discrete function, that is, a series of impulses each day: *w* ^*i*^ on day *i*, and the product of convolution as a summation in which the model performance$\;{\hat{p}}^{n}\;$on day  *n*  is estimated by mathematical recursion from the series of *w* ^*i*^ before day *n*.

#### 2.3.2. Systems Models

**Figure 1:**
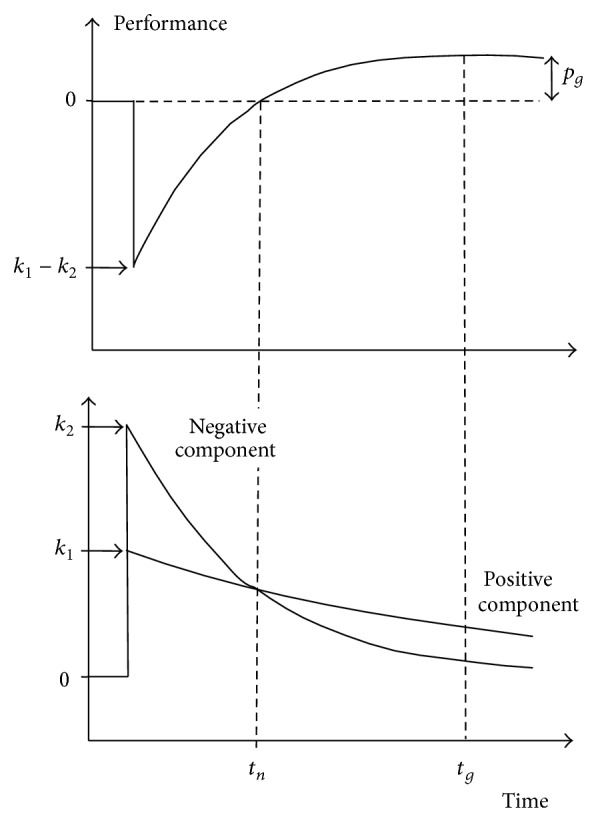
Schematic representation of the response to 1 unit of training according to Model-2Comp. Performance results from the difference between two training components. In the case where  *k*_2_  is greater than  *k*_1_, performance decreases first after the training bout. Afterwards, the negative component decreases more quickly than the positive component, in the case where  *t*_1_  is greater than  *t*_2_, resulting in performance recovery and peaking when the difference between the negative and positive components is the greatest. The response to a training bout is characterized by  *t*_*n*_, the time necessary to recover initial performance after the training session,  *t*_*g*_, the time necessary to reach maximal performance, and  *p*_*g*_, the maximal gain in performance for 1 training unit.

The most often used model initially proposed by Banister et al. [1] is named Model-2Comp in the present study (Figure 1). The system operates in accordance with a transfer function resulting from the difference between two components: one acting positively on performance ascribed to training adaptations and the second acting negatively on performance ascribed to the fatiguing effects of exercise. Responses to training are thus characterized by the set of model parameters including two gain-terms *k*_1_ and *k*_2_ and two time constants *τ*_1_ and *τ*_2_. The equation of Model-2Comp is

(6) $$\begin{array}{c} {\hat{p}}^{n}=p\left(0\right)+k_{1}\cdot \overset{n-1}{\underset{i=1}{\sum }}w^{i}\cdot e^{-\left(n-i\right)/\tau_{1}}-k_{2}\cdot \overset{n-1}{\underset{i=1}{\sum }}w^{i}\cdot e^{-\left(n-i\right)/\tau_{2}}. \end{array}$$

To assess the statistical significance of Model-2Comp, its goodness-of-fit was compared with that of a systems model comprising only one training component (Model-1Comp), whose equation is

(7) $$\begin{array}{c} {\hat{p}}^{n}=p\left(0\right)+k_{1}\cdot \overset{n-1}{\underset{i=1}{\sum }}w^{i}\cdot e^{-\left(n-i\right)/\tau_{1}}. \end{array}$$

It was shown that the fitting of performance can be significantly improved with a model with  *k*_2_  varying over time in accordance with system input [3]. We tested this model, noted here as Model-3Comp, whose equation is

(8) p^n=p0+k1·∑i=1n−1wi·e−(n−i)/τ1 −∑i=1n−1k0−Δk2i·wi·e−(n−i)/τ2

in which the value of *k*_2_ at day *i* is estimated by mathematical recursion using a first-order filter with a gain term *k*_3_ and a time constant *τ*_3_

(9) $$\begin{array}{c} {\Delta k_{2}^{i}=k}_{3}\cdot \overset{i}{\underset{j=1}{\sum }}w^{j}\cdot e^{-\left(i-j\right)/\tau_{3}}. \end{array}$$

We added the value of *k*_2_ at time 0 in this study, noted *k*_2_(0).

#### 2.3.3. Estimation of Model Parameters and Statistics

The parameters for the models were determined by fitting the model performances to actual performances for the entire group of rats using a mixed-effects model. This model incorporated a systematic component for the mean response of the population and a random component for each animal's response around the mean. The general model included (i) common time constants: *τ*_1_ for Model-1Comp, *τ*_1_ and *τ*_2_ for Model-2Comp, and *τ*_1_, *τ*_2_, and *τ*_3_ for Model-3Comp; (ii) a subject-specific intercept *p*(0); and (iii) subject-specific multiplying factors for each component: *k*_1_ for Model-1Comp, *k*_1_ and *k*_2_ for Model-2Comp, and *k*_1_, *k*_2_(0), and *k*_3_ for Model-3Comp. The set of model parameters was calculated to produce the equation that most closely fit the data points. Using the generalized reduced gradient (GRG) algorithm in the Excel solver, the parameters were determined by minimizing the residual sum of squares (RSS) between the modeled and measured performances given by

(10) $$\begin{array}{c} \text{RSS}=\overset{R}{\underset{r=1}{\sum }}\overset{N}{\underset{n=1}{\sum }}{\left(p_{r}^{n}-{\hat{p}}_{r}^{n}\right)}^{2}, \end{array}$$

where *r* is an integer corresponding to each rat (total number *R* being 11) and *n* to each day during which performance was measured (total number being 19 for each rat). *p*_*r*_^*n*^ is the actual performance and ${\hat{p}}_{r}^{n}$ is the model performance at day *n* for rat *r*.

**Figure 2:**
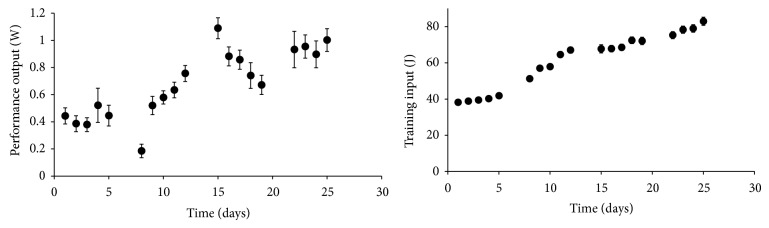
Quantification of training (systems input) and performance (systems output). Values are expressed in mean ± SEM. Note that, for the training input, the variability is very low because the animals had the same age and the same training load calculated as a percentage of body mass. Thus, SEM bars are hardly visible.

**Figure 3:**
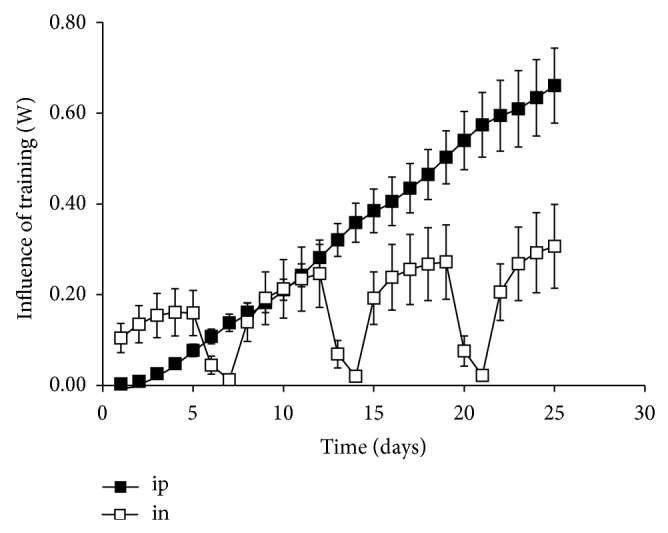
Mean ± SEM of the sum of positive and negative influences of training on performance.

Indicators of goodness-of-fit were estimated for each model used in this study. The Shapiro-Wilk test was used to check the normality of the distribution of both the training loads, that is, input of the model, and the performances, that is, input of the model. The statistical significance of the fit was tested by analysis of variance of the RSS in accordance with the degrees of freedom (df) of each model:* 12* for Model-1Comp, 24 for Model-2Comp, and* 36* for Model-3Comp. The adjusted coefficient of determination (Adj.*R* ^2^) was computed to take into account the difference in df between the models. The mean square error on performance estimation (SE) was computed as RSS/(*N* − df − 1). The level of confidence for each level of model complexity was tested by analysis of variance of the related decrease in residual variation. The decrease in RSS explained by the introduction of further model parameters was tested using the *F*-ratio test in accordance with the increase in df as described previously [15]. Data in the text and Table 1 are expressed as means ± SD and the responses to training are showed with SEM in Figures 2 and 3.

#### 2.3.4. Modeled Responses to Training

With Model-2Comp, the time response of performance to a single training bout was characterized by *t*_*n*_, the time to recover performance, and *t*_*g*_, the time to peak performance after training completion [16], computed as

(11) $$\begin{array}{c} t_{n}=\frac{\tau_{1}\tau_{2}}{\tau_{1}-\tau_{2}}\ln\left(\frac{k_{2}}{k_{1}}\right),\;\;t_{g}=\frac{\tau_{1}\tau_{2}}{\tau_{1}-\tau_{2}}\ln\left(\frac{\tau_{1}k_{2}}{\tau_{2}k_{1}}\right). \end{array}$$

*p*_*g*_, the maximal gain in performance for 1 unit of training, was estimated as

(12) $$\begin{array}{c} p_{g}=k_{1}e^{-t_{g}/\tau_{1}}-k_{2}e^{-t_{g}/\tau_{2}}. \end{array}$$

To distinguish the short-term negative effect of the training doses from the long-term benefit, the positive and negative influences of training on performance (ip and in, resp.) were estimated as described previously [17]. The amount of training on day *i* had an effect on performance on day *n* quantified by

(13) $$\begin{array}{c} E\left(\frac{i}{n}\right)=k_{1}w^{i}e^{-\left(n-i\right)/\tau_{1}}-k_{2}w^{i}e^{-\left(n-i\right)/\tau_{2}}. \end{array}$$

The values of in and ip on day *n* were estimated from the sum of influences of each past training amount, depending on whether the result was negative or positive:

(14) $$\begin{array}{c} \text{i}{\text{n}}^{n}=\overset{n-1}{\underset{i=1}{\sum }}\left|E\left(\frac{i}{n}\right)\right|,\;\text{when }E\left(\frac{i}{n}\right)<0\\ \text{i}{\text{p}}^{n}=\overset{n-1}{\underset{i=1}{\sum }}\left|E\left(\frac{i}{n}\right)\right|,\;\text{when }E\left(\frac{i}{n}\right)>0. \end{array}$$

## 3. Results

Figure 2 shows the evolution in training work and performance.  Table 2 shows the statistics for the fitting of performance with the three tested models. Although the fit was statistically significant for all models, only Model-2Comp significantly improved the fit when compared with Model-1Comp (*P* < 0.05). The third component in Model-3Comp failed to give a description of performance variations compared with Model-1Comp and Model-2Comp (*P* > 0.05). It is noteworthy that the coefficient of determination adjusted to the model df was lower for Model-3Comp than for Model-2Comp.

Because of its statistical significance, the results from Model-2Comp were retained for the analysis of the effects of training. With the estimates of parameters of Model-2Comp (*τ*_1_ = 5.31 days, *τ*_2_ = 4.3 days, *k*_1_ = 0.0186 ± 0.0134, and *k*_2_ = 0.0200 ± 0.0157 s^−1^), the response to a training bout was characterized by *t*_*n*_ = 1.07 ± 1.46 days, *t*_*n*_ = 5.29 ± 2.04 days, and *p*_*g*_ = 0.0011 ± 0.0005 W. The variations in ip and in are shown on Figure 3. ip, which can be regarded as an index of the adaptations to physical training, increased progressively all along the experiment, whereas in, the index of fatigue, increased during the first days of training each week before it plateaued with the daily sessions. The 2 days without training between weeks allowed a complete recovery of past sessions.

## 4. Discussion

In the present study, Model-2Comp was retained as the optimal model because statistically it provided the best description of the effect of the response to resistance training in rats. Contrary to the results in a previous report [3], Model-3Comp did not statistically improve the fit of the model, possibly because of an insufficient amount of training work. This model is based on the assumption that the relationship between daily training work and performance has an inverted-U shape, which implies that when the amount of training exceeds an optimal value of daily work, performance will decline because of the transient oversolicitation. The amounts of training in the present study may not have been great enough to allow the detection of such an effect. The variations in fatigue elicited by the exercise over the entire study period were relatively small and Model-3Comp did not increase the response to training compared to Model-2Comp. This is supported by the estimates for the small values obtained for *t*_*n*_ and *t*_*g*_, which suggested that the rats coped well with the training work. Nevertheless, Model-3Comp is of a great interest for exercise prescription because it allows for more detailed analysis of the detrimental effects of training with heavy/supraoptimal loads. For this reason, this preliminary study with an experimental animal model provides a basis for further research using Model-3Comp. Indeed, to optimally capture the process of training, it will be necessary to increase the amount of training work and to use contrasted training programs with periods of more intensified training followed by reduced training work. Moreover, this systematic mathematical procedure of modeling offers the possibility of simulating training effects in order to test different strategies, and it may thus be useful for advocating individualized training programs, which constitute the optimal adaptive stimulus. This type of approach was developed to optimize the training process in athletes [18, 19], but, with the animal as an experimental model, it could be extended to those chronic diseases for which exercise presents curative properties as already employed in cardiac rehabilitation [20, 21]. It would thus be of interest to extend these strategies of rehabilitation programs to rodent models suffering from other chronic diseases (e.g., ob/ob mice, db/db mice for type 2 diabetes, and the streptozotocin model for type 1 diabetes), as direct testing in patients would not be ethical.

Another advantage of the animal model compared with human modeling of training effects is the high precision in the quantification of training work and performance. In the present study, the training work was directly computed by the mechanical work of the center of mass [22]. Here, the unit was the joule, whereas the training load for athletes is indirectly evaluated by the variation in heart rate, as initially proposed by Banister, or the number of repetitions in each exercise bout [17, 23, 24]. The measure of performance is also more accurate because it is computed from the power developed according to the reference method of the center of mass [22]. This measure in each training session also allows the collection of a high number of performance values needed to fit the model.

This study is the first to blend the mixed-effects model in that proposed by Banister, that is, Model-2Comp. This advance in the technical sophistication of the modeling led us to pool the data of the entire group of animals which offers two main advantages over the classical single-individual model. The first advantage is that it provides great robustness in the determination of the model parameters and insofar it increases the number of performance criteria, without increasing the degrees of freedom of the model in the same proportion. The second advantage is that it offers the possibility of sacrificing several animals during training to gain information about the dynamics of the biological processes involved, without appreciably degrading the precision of the training response quantification. The only precaution that needs to be taken is to adapt the number of animals included in the study according to the number of biological measures planned at different times so that the training response at the end of the training period is still representative with regard to a sufficient sample size.

Last, compared with studies on training effects in athletes, the animal model offers optimal conditions to link both the positive and negative effects of training to the transitory adaptive mechanisms induced by the cell signaling pathways. Until now, the process of training adaptation was considered to be like a black box, wherein performance output is the response to training work. With an animal model that conforms to the standards for the ethical treatment of experimental animals, it is possible to give the real physiological signification to the components of the transfer function used to describe the training effects on performance. New hypotheses can thus be formulated to explain the positive and negative training effects on performance. For example, is the positive influence (ip) linked to the main protein synthesis-signaling pathway under the control of the mechanistic (or mammalian) target of rapamycin MTOR or is it related to the signaling scaffold that is responsible for morphological adaptions (phenotype, ATPase activity, and hyperplasia)? On the other hand, can the negative influence (in) be explained by exercise-induced proteolysis, a phenomenon which seems to be attenuated at least in part by resistance training through attenuated induction of atrogenes, such as the muscle ring finger 1 (MuRF-1) [25]?

## 5. Conclusion

Modeling the effects of resistance training is fully applicable in rodent and allows for the detailed analysis of the training adaptation process. Model-2Comp was the most appropriate model to describe the training responses in the present study. The addition of contrasted periods to our training program may be promising for the application of Model-3Comp, which would yield information on the optimal value of daily training work, a major focus in research on individualized training and rehabilitation programs. The mixed-effects model offers two main advantages compared with individual classical modeling, with (i) greater robustness in the determination of the model parameters and (ii) the possibility to determine the kinetic of the biological parameters by sacrificing several animals at critical times during the training program. The accuracy in quantifying training loads and performance in the experimental condition of resistance training with rodents, as well as the possibility of tightly controlling external factors, makes it possible to upgrade the structure of the training effects model and establish the biological significance of the model components.

## Figures and Tables

**Table 1:** Change in additional loads lifted by rats during the training program.

Training sessions	Load (% body mass)	Mean load ± SD (g)
1 to 5	50	143.8 ± 10.2
6	80	248 ± 20.1
7 and 8	100	312.6 ± 24.6
9 to 13	120	397.1 ± 34.7
14 to 16	130	450.9 ± 37.8
17 and 18	140	497.5 ± 41.6
19	150	539.7 ± 47.6

**Table 2:** Statistics of model fitting.

Model	*R* ^2^	Adj.*R* ^2^	F ratio	df	*P*	SE
Model-1Comp	0.48	0.45	14.97	12, 196	<0.001	0.209
Model-2Comp	0.53^*^	0.47	8.78	24, 184	<0.001	0.202
Model-3Comp	0.54	0.45	5.68	36, 172	<0.001	0.198

Model-1Comp, model using one first-order component; Model-2Comp, model using two first-order components; Model-3Comp, model with two components where the gain term for the negative component varies by using one further first-order filter. Adj.*R* ^2^, adjusted coefficient of determination; df, degrees of freedom; SE, standard error. Statistical difference compared to Model-1Comp: ^*^ *P* < 0.05.
